# BDNF Promotes EGF-Induced Proliferation and Migration of Human Fetal Neural Stem/Progenitor Cells via the PI3K/Akt Pathwa

**DOI:** 10.3390/molecules161210146

**Published:** 2011-12-06

**Authors:** Qi Zhang, Gang Liu, Yi Wu, Hongying Sha, Pengyue Zhang, Jie Jia

**Affiliations:** 1 Department of Rehabilitation, Huashan Hospital, Fudan University, Shanghai 200040, China; Email: friday0451@163.com (Q.Z.); 2 Department of Sports Medicine and Rehabilitation, Medical College of Fudan University, Shanghai 200032, China; 3 The Yonghe Branch of Huashan Hospital, Fudan University, Shanghai 200436, China; 4 State Key Laboratory of Medical Neurobiology, Fudan University, Shanghai 200032, China

**Keywords:** neural stem/progenitor cells, epidermal growth factor, brain-derived neurotrophic factor, proliferation, migration

## Abstract

Neurogenesis is a complex process, which contributes to the ability of the adult brain to function normally and adapt to diseases. Epidermal growth factor (EGF) is known to play an important role in neurogenesis; however, the underlying mechanism is still unclear. Here, we hypothesized that brain-derived neurotrophic factor (BDNF) can enhance the effect of EGF on neurogenesis. Using *in vitro* cell culture of aborted human fetal brain tissues, we investigated proliferation and migration of neural stem/progenitor cells (NSPCs) after treatment with EGF and different concentrations of BDNF. EGF stimulated proliferation and migration of NSPCs, and this effect was significantly enhanced by co-incubation with BDNF. In the NSPCs treated with 50 ng/mL BDNF, BrdU incorporation was significantly increased (from 7.91% to 17.07%), as compared with that in the control. Moreover, the number of migrating cells was at least 2-fold higher than that in the control. Furthermore, phosphorylation of Akt-1 was increased by BDNF treatment, as well. By contrast, the enhancing effect of BDNF on EGF-induced proliferation and migration of NSPCs were abolished by an inhibitor of PI3K, LY294002. These findings suggest that BDNF promotes EGF-induced proliferation and migration of NSPC through the PI3K/Akt pathway, providing significant insights into not only the mechanism underlying EGF-induced neurogenesis but also potential neuronal replacement strategies to treat brain damage.

## 1. Introduction

Neurogenesis is a complex developmental process involving proliferation, differentiation, migration, survival, maturation and functional integration of neural stem/progenitor cells (NSPCs) into neuronal circuits [1]. Adult neurogenesis in higher organisms, including primates and humans, is largely restricted to two regions: the subventricular zone (SVZ) of the lateral ventricles and the subgranular zone (SGZ) of the hippocampus. The process of neurogenesis involves a dynamic regulatory network of diverse biological molecules, including hormones, transcription factors, and neurotransmitters [2,3]. Growing evidence has indicated that growth factors, which are important for regulating a variety of cellular processes, may also play an important role in neurogenesis [4,5].

Epidermal growth factor (EGF) regulates cell growth by stimulating proliferation and migration of different types of cells. In the central nervous system (CNS), EGF mRNA has been detected in many regions, including the brainstem, cerebellum, cerebral cortex, hippocampus, olfactory bulb, and striatum. The highest levels, however, were found in the olfactory bulb, basal hypothalamus and cerebellum [6]. Intracerebral infusion of EGF resulted in a dramatic proliferation of endogenous SVZ precursor cells [7]. EGF has also been demonstrated to increase the number of newborn cells in the striatum either by stimulating migration of SVZ cells or by promoting proliferation of local progenitor cells [6]. Moreover, EGF has beenshown to stimulate the migration and proliferation of murine progenitor cells *in vivo* after transplantation to the adult rat brain [8]. Collectively, evidence has demonstrated that EGF provides important extracellular signals during development of CNS. However, EGF and its associated signaling pathways during human neurogenesis remain unclear.

Neurotrophic factors, such as brain-derived neurotrophic factor (BDNF) that contributes to neuronal survival and differentiation during development, also play fundamental roles in CNS neurogenesis. It has been shown that BDNF activates adult neurogenesis in the SVZ. Moreover, BDNF infusion led to increases in newly born neurons and SVZ neurogenesis in the olfactory bulb and SVZ [9,10]. However, administration of BDNF into the lateral ventricles resulted in a decrease in neurogenesis in the SVZ [11]. Thus, further studies are required to clarify the effect of BDNF. More recently, it has been reported that the co-administration of BDNF and EGF induce striatal neurogenesis and promote functional recovery in an adult animal model of neonatal hypoxic-ischemic brain injury [5]. Additionally, emerging evidence has shown that EGF and BDNF-induced cell proliferation and survival were mediated via the PI3K-Akt pathway [12,13]. However, it is unclear whether BDNF has a stimulatory effect on EGF-induced neurogenesis through this same pathway. Akt-1 is a well-characterized serine/threonine kinase and downstream target of PI3K, which acts as a key modulator of survival and proliferation processes in various types of cells [14]. Recent studies have demonstrated that the activation of the PI3K/Akt-1 pathway affects not only cell proliferation but also migration [1]. Thus, we hypothesized that there must be an existing regulatory network involving growth factors and neurotrophic factors during neurogenesis.

In the present study, we sought to determine whether specific neurotrophic factors were capable of enhancing the effect of EGF on neurogenesis. We investigated the effect of BDNF on the EGF-induced NSPCs proliferation and migration by using an *in vitro* human fetal brain cell culture system. LY294002, a PI3K inhibitor, was used to further analyze the mechanism underlying BDNF and EGF-induced proliferation and migration. Results showed that LY294002 was able to abolished BDNF stimulatory effect on EGF-induced NSPCs proliferation, migration and phosphorylation of Akt-1. Taken together, our findings revealed that BDNF can act as a stimulator of EGF-induced NSPC proliferation and migration via the PI3K/Akt pathway. Furthermore, our study highlighted the important functions of both growth factors and neurotrophic factors in the neurogenesis of CNS.

## 2. Results

### 2.1. Neurosphere Formation of Human NSPCs

Human NSPCs were isolated from the brain tissues of aborted human fetus. Following seeding into growth medium, we found that aggregates of dividing cells formed into neurospheres. After seven days of incubation, individual neurospheres were sectioned into 4 pieces using a fine glass needle and were respectively transferred into fresh growth medium containing bFGF2 and EGF. The pieces formed new neurospheres within the next 24 hours, eventually reaching sizes close to that of the primary neurospheres. When the neurospheres could be passaged stably over three passages, we began to passage the neurospheres using Tryple^TM^ Express for digestion. Using these methods, we efficiently obtained human NSPCs. Results of immunocytochemical staining showed these neurospheres were positive for nestin (data not shown).

### 2.2. BDNF Treatment Increased EGF-Induced Proliferation of Human NSPCs

**Figure 1:**
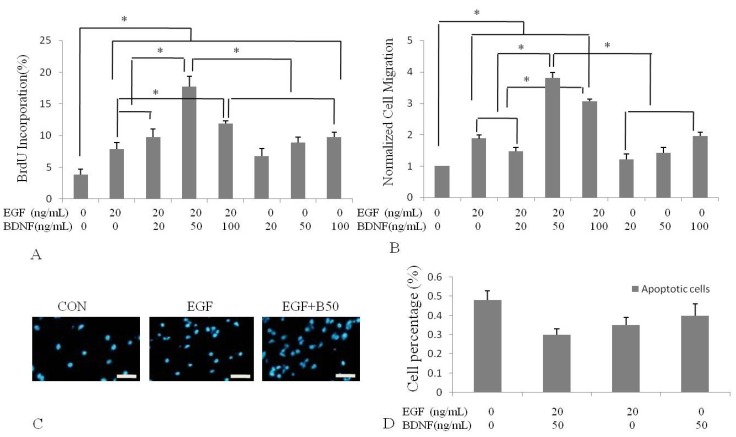
Effect of BDNF on EGF-induced human NSPC proliferation and migration. Quantitative analysis of human NSPCs proliferation (**A**) and migration (**B**) were determined by BrdU incorporation and Transwell assay following 2 hours treatment of different concentrations of BDNF stimulation with 20 ng/mL EGF or not. (B) Data were expressed as mean ratio compared with the control group; (**C**) Cells incubated with EGF or EGF and BDNF. Nuclei were counter-stained with DAPI; (**D**) Analysis of apoptosis by Flow-cytometry. There was no appreciable change in these groups CON: control (incubated with medium without EGF or BDNF). * *p *< 0.05, Scale bar = 20 μm.

To characterize the effect of BDNF on EGF-induced proliferation of NSPC, we incubated human NSPCs in various concentrations of BDNF (0, 20, 50 and 100 ng/mL) alone or with 20 ng/mL EGF and using a well-established *in vitro* NSPC culture system. The cells were treated in the absence of bFGF2 to avoid any interfering effect. The capacity of EGF to promote NSPC proliferation was evaluated by BrdU incorporation. Quantification data showed that, 7.91%, 6.95%, 8.90% and 9.73% of NSPCs proliferated when they were treated separately with 20 ng/mL EGF, 20 ng/mL, 50 ng/mL and 100 ng/mL of BDNF. The percentage of proliferating NSPCs increased to 9.74% upon treatment with 20 ng/mL EGF and 20 ng/mL BDNF, to 17.07% with EGF and 50 ng/mL BDNF, and to 11.92% with EGF and 100 ng/mL BDNF. These results demonstrated that EGF-activated NSPC prwoliferation was stimulated by BDNF. The highest level of proliferation was found at 50 ng/mL BDNF, which was nearly 2-fold than achieved with the 20 ng/mL BDNF dosage. The highest BDNF dosage (100 ng/mL) led to slightly less proliferation (Figure 1A,C).

To decide whether EGF or BDNF increases cell number by increasing proliferation or decreasing apoptosis, we measured apoptotic cells number by flow cytometry. We found that treating cells with EGF or BDNF resulted in a decrease in the percentage of apoptotic cells, however there was no appreciable change in these groups (Figure 1D). Thus, EGF and BDNF increased human the number of NSPCs primarily by increasing cell proliferation.

### 2.3. BDNF Treatment Increased EGF-Induced Human NSPC Migration

Next, to determine whether BDNF alters EGF-induced migratory potential of NSPCs, we performed Transwell assays. NSPCs were treated in BDNF (0, 20, 50 and 100 ng/mL) combined with 20 ng/mL EGF or alone, and the effect on NSPC migration was confirmed by counting the number of migrating cells. The results of DAPI nuclear staining revealed that EGF-driven migration was enhanced by BDNF treatment. When compared with the EGF group, the number of migrating cells reached 0.78-fold by exposure to EGF and 20 ng/mL BDNF (Figure 1B). The amount of migrating cells continued to increase, by at least 2-fold, at the concentration of EGF and 50 ng/mL BDNF, which was much higher than treated with various concentrations of BDNF and represented the maximal migrating effect observed in our study. Moreover, the number of migrating cells treated with EGF and 50 ng/mL BDNF was also increased, by nearly 1.2-fold over the amount induced by treatment with EGF and 20 ng/mL BDNF, indicating that BDNF increased migration of NSPCs in a dose-dependent manner. However, the number of migrating cells started to slightly decrease, to 1.63-fold over that of EGF group, at the concentration of 100 ng/mL.

### 2.4. BDNF Promotes EGF-Induced NSPC Proliferation and Migration via the PI3K/Akt Kinase Pathway

**Figure 2:**
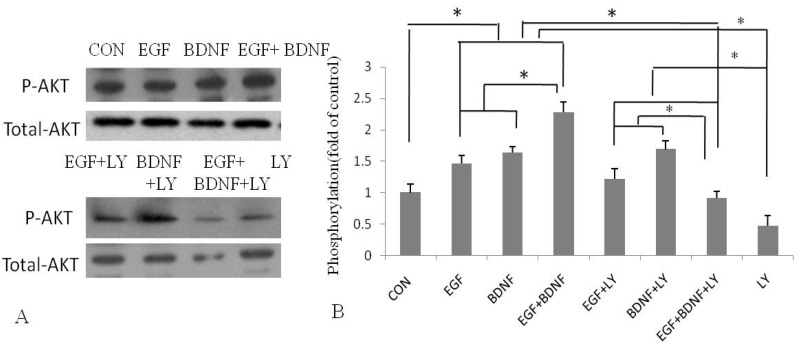
Western blot analysis phosphorylation and expression of AKT. NSPCs were pretreated with LY294002 (20 μM) for 2 hours before exposure to EGF (20 ng/mL) and BDNF (50 ng/mL). (**A**) Western blotting analysis showed increased and decreased activation (phosphorylation) of Akt-1 with BDNF stimulation in NSPCs pretreated with or without LY294002, respectively; (**B**) Data are expreseed as the ratio of phosphorylated to total Akt-1. * *p* < 0.05.

The above results indicated that BDNF had the capacity to stimulate EGF-induced NSPCs proliferation and migration with the peak effect observed at the concentration of 50 ng/mL BDNF. To further elucidate the mechanisms underlying BDNF accelerating proliferation and migration of NSPC induced by EGF, we first investigated the association between BDNF and phosphorylation of Akt-1 by Western blotting. Results showed that 50 ng/mL BDNF significantly increased phosphorylation of Akt-1 in NSPCs by 2-fold (Figure 2), as compared with the control.

**Figure 3:**
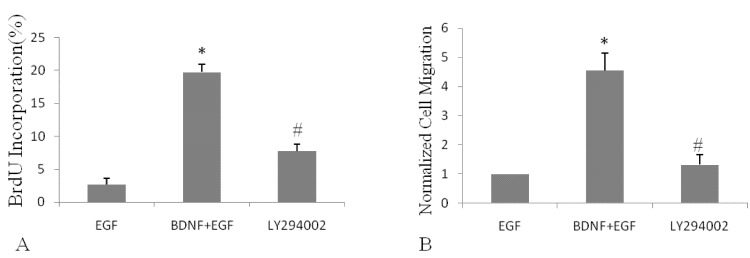
Effects of BDNF on EGF-mediated human NSPC proliferation and migration were abolished by pretreatment with LY294002.EGF: Cells incubated with 20 ng/mL EGF. EGF+BDNF: cells incubated with 20 ng/mL EGF and 50 ng/mL BDNF. LY294002: Cells incubated with LY294002 before exposure to 20 ng/mL EGF and 50 ng/mL BDNF. * *p *< 0.05, as compared with the EGF group; # *p *< 0.05, as compared with the BDNF+EGF group.

Next, we analyzed the involvement of the PI3K/Akt pathway in the proliferation and migration stimulated by BDNF. NSPCs were pretreated with LY294002, a specific PI3K inhibitor, before incubation with EGF and BDNF. The results showed that in the presence of LY294002, the proliferation of NSPCs was strongly inhibited, by nearly 3-fold, and the number of migrating cells was also significantly decreased, also by 3-fold (Figure 3). These results were coincident with those obtained from Western blotting analysis of phosphorylation of Akt-1 (Figure 2). Taken together, these results suggested that the PI3K/Akt pathway is involved in BDNF stimulation of EGF-induced proliferation and migration of NSPCs.

## 3. Discussion

In the present study, we have demonstrated the direct effect of BDNF on EGF-induced proliferation and migration of NSPCs using an *in vitro* culture system. We isolated and cultured human fetal brain NSPCs and showed that 50 ng/mL of BDNF produced the maximal effect on EGF-induced NSPC proliferation and migration. Furthermore, we have demonstrated that BDNF increased the phosphorylation of Akt-1, a downstream target of PI3K. However, this enhanced effect was abolished when NSPCs were pretreated with the PI3K inhibitor LY294002. These results indicated that BDNF is involved in EGF-induced proliferation and migration during neurogenesis *via* regulation of the PI3K/Akt pathway. It has been shown that adult brain injuries such as trauma and ischemia induce neurogenesis, providing hope for functional recovery after a CNS insult [15,16]. Although postnatal neurogenesis can occur, its capacity is considerably limited and regeneration of new neurons normally occurs only in two regions of the adult brain, namely the hippocampus and olfactory bulbs [17,18,19]. Thus, transplantation of NSPCs at sites of brain injury may be a better way to promote functional recovery. On the other hand, growth factors required for neuronal proliferation and differentiation could be used in combination with NSPCs transplantation as a potential treatment for neurodegenerative disorders or brain damage. 

Many studies have shown that EGF plays important roles in the regulation of cell proliferation, migration, and differentiation [20,21,22,23]. Infusion of EGF and FGF2 has been shown to significantly increase proliferative rate and differentiation of endogenous NSPCs following ischemic injury [24,25]. Furthermore, EGF has been shown to promote the *in vitro* differentiation of neural crest cells to neurons and melanocytes [26]. However, one recent study has shown that EGF-induced neurogenesis was not effective, as manifested by lack of a sustained increase of BrdU-positive cells at four weeks after trauma, indicating that EGF needs to be combined with other factors to accelerate proliferation of neural cells [27]. BDNF is a member of the neurotrophin family, which may play important roles during neuronal development and throughout the life of the animal. During environmental induction of hippocampal neurogenesis in rodents, the expression of BDNF is found to be increased significantly [28]. BDNF may act subsequently as a mitogens like EGF in the differentiation of newly generated neurons derived from stem cell precursors. Recently, other evidence has shown that BDNF and EGF were capable of inducing neurogenesis and promoting functional recovery after brain injury [5] and cell migration in human Caov3 cells [12].

In our study, we detected that BDNF enhanced EGF-induced proliferation and migration rate of NSPCs. 50 ng/mL of BDNF elicited the maximal effect, which was not affected by apoptosis rate. However, compare to 50 ng/mL of BDNF, a further increase in the concentration of BDNF (100 ng/mL) induced slightly less proliferation and migration, which may be due to the effect of enhancing differentiation of neuronal precursors. Further studies should be done to confirm this speculation. The result indicated that a certain range of concentration BDNF enhances EGF-induced cell proliferation and migration; beyond the range affect reduced or have opposite effect. Treated with appropriate dose of BDNF may significantly improve the effect of EGF-dependent NSPCs to control neurodegenerative diseases or injury.

Proteins AKT, ERK-1/2 and STAT-3 are involved in many important physiological processes. Furthermore, these three signaling proteins are pivotal switches that mediate the neurogenesis stimulated by cytokines. In this study, we found that in the presence of PI3K inhibitor, the NSPCs proliferation and the numbers of migrating cells were strongly inhibited, however, the levers was still higher than that of the EGF group. These results indicated that the PI3K/Akt pathway is involved in the stimulation of BDNF in EGF-induced proliferation and migration of NSPCs, which provides some insight into the mechanisms underlying the regulatory roles of BDNF and EGF in neurogenesis. Nevertheless, further study is necessary to clarify the interaction between BDNF and NSPCs, such as effect of BDNF on the expression of neuronal markers and certain signaling pathways activated by BDNF in NSPCs.

## 4. Experimental

All experiments were approved by the Committee for Human Research Subjects of Huashan Hospital (Shanghai, China). Recombinant EGF and BDNF were obtained from Invitrogen (Carlsbad, CA, USA), and LY294002 was purchased from Calbiochem (San Diego, CA, USA).

### 4.1. Primary Cell Culture Preparation

Cells were prepared as described previously [29]. Briefly, human NSPCs were prepared from 13-week fetus cortex of selectively terminated normal pregnant women. Informed consent was obtained before specimen collection. The cortex was dissected in chilled sterile phosphate buffered saline containing 0.6% glucose. The tissues were triturated in 0.1% trypsin containing 0.04% DNase using a fine polished Pasteur pipette, followed by incubation in the same solution for 20 minutes (min) at 37 °C. The cells were seeded into substrate-free tissue culture flasks at a concentration of 2 × 10^5^/mL and cultured in DMEM/F12 media containing 50 units/mL penicillin-streptomycin, B27 supplement (1:50; Invitrogen), bFGF-2, EGF (both at 20 ng/mL), and heparin (5 μg/mL). Passaging was performed for three times using a sectioning method [30], and subsequent passaging was carried out using TrypleTM Express (Invitrogen).

### 4.2. Proliferation Assay

Before each experiment, NSPCs were passaged and seeded in 24-well plates (coated with poly-D-lysine, 10 μg/mL) at a density of 4 × 10^4^ cells/well in culture medium for 24 hours. The medium without growth factors was replaced with medium supplemented with different concentrations of BDNF (0, 20, 50 and 100 ng/mL) alone or with EGF (20 ng/mL) and cells were incubated for 2 hours. For the experiment utilizing inhibitor, the cells were pretreated with LY294002 (20 μM) for 2 hours [1], followed by incubation with 50 ng/mL BDNF for 2 hours.

### 4.3. Flow Cytometry Assay

The method used was described previously [1]. Briefly, NSPCs were treated with or without BDNF. Single cells were harvested by trypsin digestion and mechanical disassociation. Cell proliferation was examined by BrdU staining. For the BrdU incorporation assay, NSPCs were treated with BrdU (100 mg/mL; Sigma-Aldrich, St. Louis, MO, USA) for 24 hours and then stained with FITC-BrdU (20 μL/10^6^ cells; BD Biosciences, San Diego, CA, USA). Apoptotic cells death was determined using an FITC-Annexin V/PI Apoptosis Detection Assay according to the manufacturer’s protocol (Beijing Biosea Biotechnology, China). All data were obtained and analyzed by flow cytometry using the Cell Quest software on a FACScan instrument (BD Biosciences).

### 4.4. Transwell Cell Migration Assay

The method used was as described previously [31,32]. Briefly, all migration assays were performed in triplicate wells using 6.5-mm Transwell chambers with 8-μm pore size (Corning Costar, Lowell, MA, USA). NSPCs in suspension (100 μL, 2 × 10^5^ in medium) were mixed with various concentrations of BDNF (0, 20, 50 and 100 ng/mL) alone or with EGF and added to the upper chamber. All lower chambers were filled with 650 μL of medium, and the plates were incubated at 37 °C for 24 hours. In inhibition experiments, the cells were pretreated for 2 hours at 37 °C with LY294002 before 50 ng/mL BDNF and EGF addition. After incubating, the remaining cells in the upper chamber were removed. The filter was stained with DAPI (1 μg/mL). The cells were counted under a microscope and the number of migrating cells per well was calculated by averaging the number of cells counted in three separate high power fields. The counts for three replicate wells were averaged.

### 4.5. Western Blotting

To examine the effect of BDNF on the phosphorylation of Akt, cells were cultured in medium with EGF and BDNF (0, 20, 50 and 100 ng/mL) or treated with BDNF alone for 2 hours. For experiments utilizing the inhibitor LY294002, cells were pretreated with LY294002 (20 μM) for 2 hours and then stimulation with 50 ng/mL BDNF and EGF for 2 hours. For the analysis of phosphorylated proteins, the cells were lysed in lysis buffer supplemented with phosphatase inhibitors (20 mM β-glycero phosphate, 10 mM NaF, and 0.1 mM Na_3_VO_4_). Equal amounts of proteins were separated on a 10% SDS-polyacrylamide gel by electrophoresis and then transferred onto PVDF membranes by western blotting. After blocking of non-specific binding, the membrane was incubated with primary antibodies for phosphorylated Akt-1 and total Akt-1 (1:1,000; Cell Signaling Technologies, Danvers, MA, USA) overnight at 4 °C, followed by incubation with horseradish peroxidase-conjugated secondary antibodies (1:10,000; Cell Signaling Technologies). Immunoreactivity was visualized by using the Enhanced Chemiluminescent (ECL) solution (Pierce, Rockford, IL, USA) and exposure to film. For data quantitative analysis, the films were scanned and band densitometry was performed with the Image J program.

### 4.6. Statistical Analyses

Data were expressed as means ± standard deviation (SD). Statistical tests for difference of means between two independent groups were performed using the two-sided Student’s *t-*test. Significance was indicated by a *p*-value of less than 0.05. All experiments were performed in quadruplicate and repeated at least three times.

## 5. Conclusions

Overall, we have provided evidence that EGF can stimulate proliferation and migration of human NSPCs. Its regulation may be affected by BDNF *via* the PI3K/Akt kinase pathway; thus, both BDNF and EGF may functionally modulate the regulatory network of neurogenesis. In addition, our results are relevant for defining the ideal culture medium for neural cells expansion *in vitro*. These results will contribute to our understanding of mechanisms underlying growth factors in the development of NSPCs during neurogenesis, and will likely be helpful for development of therapeutic applications using NSPCs to improve lifespan and integration of pre-treated cells after transplantation.

## References

[1] Wu Y., Peng H., Cui M., Whitney N.P., Huang Y., Zheng J.C. (2009). CXCL12 increases human neural progenitor cell proliferation through Akt-1/FOXO3a signaling pathway. J. Neurochem..

[2] Liu C., Zhao X. (2009). MicroRNAs in adult and embryonic neurogenesis. Neuromol. Med..

[3] Leker R.R., Lasri V., Chernoguz D.J. (2009). Neural Transm Growth factors improve neurogenesis and outcome after focal cerebral ischemia. J. Neural Transm..

[4] Türeyen K., Vemuganti R., Bowen K.K., Sailor K.A., Dempsey R.J. (2005). EGF and FGF-2 infusion increases post-ischemic neural progenitor cell proliferation in the adult rat brain. Neurosurgery.

[5] IM S.H., Yu J.H., Park E.S., Lee J.E., Kim H.O., Park K.I., Kim G.W., Park C.I., Cho S.R. (2010). Induction of striatal neurogenesisenhances functional recovery in an adult animal model of neonatal hypoxic-ischemicbrain injury. Neuroscience.

[6] Wong R.W., Guillaud L. (2004). The role of epidermal growth factor and its receptors in mammalian CNS. Cytokine Growth Factor Rev..

[7] Gonzalez-Perez O., Quiñones-Hinojosa A. (2010). Dose-dependent effect of EGF on migration and differentiation of adult subventricular zone astrocytes. Glia.

[8] Fricker-Gates R.A., Winkler C., Kirik D., Rosenblad C., Carpenter M.K., Björklund A. (2000). EGF infusion stimulates the proliferation and migration of embryonic progenitor cells transplanted in the adult rat striatum. Exp. Neurol..

[9] Zigova T., Pencea V., Wiegand S.J., Luskin M.B. (1998). Intraventricular administration of BDNF increases the number of newly generated neurons in the adult olfactory bulb. Mol. Cell. Neurosci..

[10] Shetty A.K., Turner D.A. (1998). *In Vitro* Survival and Differentiation of Neurons Derived from EpidermalGrowth Factor-Responsive Postnatal Hippocampal Stem Cells: Inducing Effects of Brain-Derived Neurotrophic Factor. J. Neurobiol..

[11] Galvao R.P., Garcia-Verdugo J.M., Alvarez-Buylla A. (2008). Brain-derived neurotrophic factor signaling does not stimulate subventricular zone neurogenesis in adult mice and rats. J. Neurosci..

[12] Qiu L., Zhou C., Sun Y., Di W., Scheffler E., Healey S., Kouttab N., Chu W., Wan Y.  (2006). Crosstalk between EGFR and TrkB enhances ovarian cancer cell migration and proliferation. Int. J. Oncol..

[13] Gschwind A., Fischer O.M., Ullrich A. (2004). The discovery of receptor tyrosine kinases: Targets for cancer therapy. Nat. Rev. Cancer.

[14] Shioda N., Han F., Fukunaga K. (2009). Role of Akt and ERK signaling in the neurogenesis following brain ischemia. Int. Rev. Neurobiol..

[15] Horner P.J., Gage F.H. (2000). Regenerating the damaged central nervous system. Nature.

[16] Wiltrout C., Lang B., Yan Y., Dempsey R.J., Vemuganti R. (2007). Repairing brain after stroke: A review on post-ischemic neurogenesis. Neurochem. Int..

[17] Hagg T. (2005). Molecular regulation of adult CNS neurogenesis: An integrated view. Trends Neurosci..

[18] Yang Z., You Y., Levison S.W. (2008). Neonatal hypoxic/ischemic brain injury induces production of calretinin-expressing interneurons in the striatum. J. Comp. Neurol..

[19] Grote H.E., Hannan A.J. (2007). Regulators of adult neurogenesis in the healthy and diseased brain. Clin. Exp. Pharmacol. Physiol..

[20] Gonzalez-Perez O., Romero-Rodriguez R., Soriano-Navarro M., Garcia-Verdugo J.M., Alvarez-Buylla A. (2009). Epidermal growth factor induces the progeny of subventricular zone type B cells to migrate and differentiate into oligodendrocytes. Stem Cells.

[21] Aguirre A., Rubio M.E., Gallo V. (2010). Notch and EGFRpathwayinteractionregulatesneuralstemcellnumber and self-renewal. Nature.

[22] Aguirre A., Rizvi T.A., Ratner N., Gallo V. (2005). Overexpression of the epidermal growth factor receptor confers migratory properties to nonmigratory postnatal neural progenitors. J. Neurosci..

[23] Gonzalez-Perez O., Alvarez-Buylla A. (2011). Oligodendrogenesis in the subventricular zone and the role of epidermal growth factor. Brain Res. Rev..

[24] Schwindt T.T., Motta F.L., Gabriela F.B., Cristina G.M., Guimarães A.O., Calcagnotto M.E., Pesquero J.B., Mello L.E. (2009). Effects of FGF-2 and EGF removal on the differentiation of mouse neural precursor cells. An. Acad. Bras. Cienc..

[25] Sun D., Bullock M.R., Altememi N., Zhou Z., Hagood S., Rolfe A., McGinn M.J., Hamm R., Colello R.J. (2010). The Effect of Epidermal Growth Factor in the Injured Brain after Trauma in Rats. J. Neurotrauma.

[26] Garcez R.C., Teixeira B.L., dos Santos Schmitt S., Alvarez-Silva M., Trentin A.G. (2009). Epidermal growth factor (EGF) promotes the *in vitro* differentiation of neural crest cells to neurons and melanocytes. Cell. Mol. Neurobiol..

[27] Johanson C., Stopa E., Baird A., Sharma H. (2011). Traumatic brain injury and recovery mechanisms: Peptide modulation of periventricular neurogenic regions by the choroid plexus-CSF nexus. J. Neural Transm..

[28] Kuzumaki N., Ikegami D., Tamura R., Hareyama N., Imai S., Narita M., Torigoe K., Niikura K., Takeshima H., Ando T. (2011). Hippocampal Epigenetic Modification at the Brain-Derived Neurotrophic Factor Gene Induced by an Enriched Environment. Hippocampus.

[29] Sha H.Y., Chen J.Q., Chen J., Zhang P.Y., Wang P., Chen L.P., Cheng G.X., Zhu J.H. (2009). Fates of donor and recipient mitochondrial DNA during generation of interspecies SCNT-derived human ES-like cells. Cloning Stem Cells.

[30] Svendsen C.N., TerBorg M.G., Armstrong R.J., Rosser A.E., Chandran S., Ostenfeld T., Caldwell M.A. (1998). A new method for the rapid and long term growth of human neural precursor cells. J. Neurosci. Methods.

[31] Bennett H.L., Brummer T., Jeanes A., Yap A.S., Daly R.J. (2008). Gab2 and Src co-operate in human mammary epithelial cells to promote growth factor independence and disruption of acinar morphogenesis. Oncogene.

[32] Kornberg L.J., Grant M.B. (2007). Adenoviruses increase endothelial cell proliferation, migration, and tube formation: Partial reversal by the focal adhesion kinase inhibitor, FRNK. Microvasc. Res..

